# Cardiometabolic risk in adolescents: prevalence and associated factors from a population-based survey (2008–2015)

**DOI:** 10.1016/j.jped.2026.101536

**Published:** 2026-04-09

**Authors:** Laura A. Bertoni, João Valentini Neto, Aline V.M. Cesar, Catharina E. Telles, Jaqueline L. Pereira, Mauro Fisberg, Regina M. Fisberg

**Affiliations:** a Universidade de São Paulo (USP), São Paulo, SP, Brazil; b Universidade Paulista (UNIP), São Paulo, SP, Brazil; c Universidade de São Paulo (USP), Faculdade de Saude Pública, Sao Paulo, SP, Brazil; d Universidade Federal de São Paulo (UNIFESP), Departamento de Pediatria, São Paulo, SP, Brazil; e School of Arts, Sciences and Humanites of University of São Paulo, São Paulo, Brazil; f Pensi Institute, São Paulo, Brazil

**Keywords:** Cardiometabolic risk factors, Adolescents, Chronic non-communicable diseases

## Abstract

**Objective:**

To investigate lipid profile alterations and their associated sociodemographic, anthropometric, and lifestyle factors among adolescents living in São Paulo in 2008 and 2015.

**Methods:**

The authors analyzed data from the ISA-Capital study, a population-based cross-sectional survey, including 448 adolescents (12–19 years). Socioeconomic, anthropometric, lifestyle, and biochemical data were collected. Cardiometabolic risk factors assessed included high blood pressure, obesity, increased waist circumference, dyslipidemia, and lipid profile alterations. Statistical analyses included Chi-square tests, *t*-tests/Wilcoxon tests, and logistic regression models (*p* < 0.05**).**

**Results:**

Dyslipidemia was the most prevalent risk factor (67.6%; 95% CI 63.1–71.8), with low HDL-c affecting 52.4% (95% CI 47.7–57.0). High blood pressure and obesity were observed in 16.1% (95% CI 12.9–19.7) and 11.7% (95% CI 9.0–15.1) of adolescents, respectively. 7% had 3 or more risk factors. Compared to 2008, adolescents in 2015 were twice as likely to present high blood pressure (OR = 2.04; *p* = 0.011). Older adolescents had lower odds of obesity (OR = 0.83; *p* = 0.024) but were more likely to have increased waist circumference (OR = 1.22; *p* = 0.027) and high blood pressure (OR = 1.17; *p* = 0.017). Adolescents self-declared as non-white had lower odds of dyslipidemia (OR = 0.59; *p* = 0.018) and high triacylglycerol (OR = 0.58; *p* = 0.012). Higher household education was associated with lower risk of presenting dyslipidemia (OR = 0.54; *p* = 0.004). Physically active adolescents had lower odds of high LDL-c (OR = 0.48; *p* = 0.045).

**Conclusion:**

The elevated prevalence of cardiometabolic risk factors, particularly dyslipidemia, underscores the need for targeted preventive strategies among adolescents in São Paulo.

## Introduction

Cardiometabolic risk is defined as the coexistence of risk factors, such as cardiometabolic alterations, that increase the risk of developing chronic conditions, such as type 2 diabetes mellitus (DM2) and CVD [1,2]. Obesity, central obesity, high serum glucose levels, high blood pressure, and dyslipidemia are examples of these cardiometabolic risk factors (CMRF) [3].

In 2017, the American Academy of Pediatrics (AAP) recommended assessing cardiometabolic risk by grouping these factors to encourage joint approaches and provide lasting benefits [4,5]. According to the NCEP-ATP III criteria, cardiometabolic risk is defined by the presence of three or more components among four categories: body composition (obesity and increased waist circumference), elevated blood pressure, altered glucose metabolism (fasting blood glucose, fasting insulinemia, and HOMA-IR index), and altered lipid metabolism (elevated total cholesterol, low HDL-c, high LDL-c, and high triacylglycerol) [6].

In 2020, the global prevalence of cardiometabolic risk factors (CMRF) was 4.8 %, affecting about 35.5 million adolescents aged 13 to 17 years [1]. The Study of Cardiovascular Risks in Adolescents (ERICA- 73,339 students) found a prevalence of 2.6 % in Brazil, 2.4 % in the Southeast, and 2.1 % in São Paulo. Despite lower prevalence rates compared to global figures, the presence of at least one risk factor is high among Brazilian adolescents, with low HDL-cholesterol affecting nearly 47 %, increasing the likelihood of health complications and further risk factors [7].

Beyond their immediate metabolic implications, lipid abnormalities during adolescence are clinically relevant because they persist into adulthood and contribute to the early development of atherosclerosis. Evidence suggests that low HDL-c and elevated LDL-c in youth are associated with endothelial dysfunction, increased arterial stiffness, and subclinical cardiovascular damage later in life [1,7].

From a social perspective, adolescence is a period marked by increased autonomy in food choices, greater exposure to unhealthy foods, sedentary behaviors, and social inequalities that shape health trajectories [8]. In middle-income countries such as Brazil, rapid urbanization and nutrition transition have intensified these exposures, disproportionately affecting socially vulnerable groups [4,7].

The development and presence of cardiometabolic risk factors (CMRF) in adolescents are linked to health risk behaviors influenced by factors like friendships, fashion trends, and impulsiveness in seeking new experiences [7]. These modifiable risk factors include short sleep duration, excessive screen time, tobacco and alcohol use, poor diet quality, and low physical activity levels [4]. Despite national studies on cardiometabolic risk in adolescents, there is limited population-based evidence on temporal changes in lipid profiles and their associated factors at the municipal level, particularly in large urban centers such as São Paulo. This study seeks to address this gap by comparing lipid alterations and related factors in adolescents across two time points.

This study investigates alterations in the cardiometabolic lipid profile and their associated sociodemographic, anthropometric, and lifestyle factors among non-institutionalized adolescents in São Paulo, comparing data from 2008 to 2015 to monitor and identify these factors and their impact on adolescent health.

## Methods

The authors used data from the ISA-Capital, a population-based, cross-sectional study that aims to investigate the health status, lifestyle, and use of health services among individuals living in the urban area of the municipality of São Paulo — SP, conducted in 2003, 2008, and 2015. The ISA-Capital study used a complex, multistage probabilistic sampling design, stratified by geographic area and census tracts. Households were randomly selected, and all eligible adolescents residing in the selected households were invited to participate. Sampling weights were applied to account for the complex design and ensure representativeness of the adolescent population of São Paulo. Data collection and procedures have been previously described in a publication [8]. The final sample of this study included 157 adolescents from ISA-2008 and 291 from ISA-2015, totaling 448 individuals.

The data used were sex (male and female), age in complete years (12 to 19 years), and self-declared color or race, classified as 'white' and 'non-white' (including Black, Brown, Indigenous, and Yellow). Income data were analyzed using the variable "per capita family income per equivalent adult," calculated by summing the monetary income reported by all adult family members, dividing by the number of adult family members, and expressing the result in Reais. The education level of the head of household was measured and categorized into two categories: “up to 9 years” (completing elementary school or less) and “10 years or more” (above completing elementary school).

In 2008 and 2015, the ISA-Nutrition data collection on food consumption used a 24-hour recall (R24h), with the first conducted in person and the second by phone. Data collection notes were made using the Nutrition Data System for Research software 2021. The nutritional value of energy and macronutrients was compared with national food composition tables, such as TACO from the Center for Food Studies and Research and the Brazilian Food Composition Data Network.

Diet quality was evaluated using the Diet Quality Index for the Brazilian population (IQD-R), proposed by Fisberg et al. (2004). The maximum score on the index is 100 points, and the higher the score, the better the diet quality [9,10].

Physical activity was measured using the International Physical Activity Questionnaire (IPAQ) long version [10]. The level of physical activity was classified according to the WHO proposal (2020) [11]. and the calculations were based on the IPAQ Data Processing and Analysis Guide, updated in 2015. The IPAQ long version used to assess physical activity has been previously validated for the Brazilian population, demonstrating adequate reliability and reproducibility [10].

Anthropometric and blood pressure measurements were taken according to standardized international protocols, with duplicate measurements to minimize random error. Biochemical analyses were performed in certified laboratories using standardized enzymatic methods, ensuring analytical reliability.

Data on weight, height, and waist circumference (WC) were collected during the second home visit of ISA-Nutrition in 2008 and 2015, following the standard measurement procedures recommended by the World Health Organization and the Food and Nutrition Surveillance System (SISVAN). All measurements were taken barefoot, in light clothing, and in duplicate. A third measurement was taken when the first two showed a difference of >5 %.

Weight (in kilograms [kg]) and height (in meters [m]) were used to calculate each participant's Body Mass Index (BMI). Subsequently, nutritional status was classified as 'within the recommended range' (BMI-for-age z-score ≤ +2 SD) and 'above the recommended range' (BMI-for-age z-score > +2 SD), according to WHO growth standards [12].

Central obesity (centimeters [cm]) was defined as “within the recommended range” (12 to 17 years < 95th percentile; 18 to 19 years: female WC ≤ 80 cm, male WC ≤ 90 cm) and “above the recommended range” (12 to 17 years ≥ 95th percentile; 18 to 19 years: female WC > 80 cm, male WC > 90 cm) [12,13].

Two blood pressure (BP) measurements were taken according to the protocol established by the VI Brazilian Guidelines for Hypertension of the Brazilian Society of Cardiology (SBC, 2010). In cases where a difference of >10 % existed between the measurements, a third measurement was taken10. Systolic and diastolic pressures were expressed in mmHg. Blood pressure was categorized as 'within the recommended range' (< 120/80 mmHg) and 'above the recommended range' (≥ 120/80 mmHg) for all adolescents aged 12 to 19 years [9,14].

In both the 2008 and 2015 editions of the ISA-Nutrition study, nursing technicians collected approximately 30 mL of blood from participants during their second home visit, following standardized procedures. Participants were instructed in advance to fast for 12 h, maintain a usual diet, avoid alcohol for 72 h, and refrain from intense physical activity the day before and the day of blood collection. The procedures adopted are detailed in a previous publication [8].

The biochemical markers were categorized as follows: Total cholesterol: ≤ 170 mg/dL ('within the recommended range') and > 170 mg/dL ('above the recommended range'); low-density lipoproteins (LDL-c): ≤ 110 mg/dL ('within the recommended range') and > 110 mg/dL ('above the recommended range'); high-density lipoproteins (HDL-c): > 45 mg/dL ('within the recommended range') and ≤ 45 mg/dL ('below the recommended range'); plasma triacylglycerol (TG): ≤ 90 mg/dL ('within the recommended range') and > 90 mg/dL ('above the recommended range') [[15], [16], [17]]. The present study did not evaluate Type 2 Diabetes Mellitus, as glycemia, insulinemia, or HOMA-IR data were not available for adolescents in ISA-Nutrition 2008.

Participants were considered to have cardiometabolic risk factors (CMRF) when they met three or more of the following conditions, as proposed by the NCEP-ATP III (National Cholesterol Education Program Adult Treatment Panel III) [18]. diagnostic criteria: elevated blood pressure, obesity, dyslipidemia, and type 2 diabetes mellitus. Based on the number of conditions, participants were divided into two groups: CMRF < 3 (<3) and CMRF ≥ 3 (greater than or equal to 3).

For the descriptive analyses, continuous variables were tested for normality using the Kolmogorov-Smirnov test. Variables with parametric distribution are presented as means and standard deviations, and those with nonparametric distribution are presented as medians and interquartile ranges (IR). Categorical variables are described in terms of absolute and relative frequencies, along with 95 % confidence intervals (CIs). Given the characteristics of the data, continuous variable comparisons were assessed using the Student’s *t*-test for normally distributed data and the Wilcoxon rank-sum test for non-parametric distributions, while categorical associations were evaluated via the chi-square test. Associations were investigated using logistic regression models, both in crude and multiple models. Analyses used STATA 14.0 in survey mode to account for complex sampling. The significance level adopted in this study was 5 % (*p* < 0.05).

The Research Ethics Committee of the School of Public Health of the University of São Paulo (CAAE no 72,940,623.4.0000.5421) approved this study.

## Results

This study aimed to investigate cardiometabolic risk factors and their associations with lifestyle aspects (dietary intake and physical activity) and sociodemographic characteristics of adolescents in São Paulo in 2008 and 2015. It also examined differences between the two years across sex, age, and sociodemographic factors. The study population (*n* = 448) was primarily composed of males (52.7 %), non-white individuals (55.2 %), and heads of household with 9 years or less of schooling (50.3 %). No differences were observed in these characteristics between the two editions of the ISA (*p* > 0.05) (Table 1).

**Table 1:** Demographic, quality of life and biochemical characteristics, according to year of study of the ISA adolescent population. São Paulo, Brazil.

Variables	ISA 2008		ISA 2015		ISA 2008 + ISA 2015	
	*n* = 157		*n* = 291		*n* = 448	
	n ( %)	CI95 %	n ( %)	CI95 %	n ( %)	CI95 %
Sex						
Male	86 (54.8)	46.9–62.5	150 (51.5)	45.8–57.3	236 (52.7)	48.0–57.3
Female	71 (45.2)	37.5–53.1	141 (48.5)	42.7–54.2	212 (47.3)	42.7–52.0
Individual's self-declared color/race						
White	79 (50.3)	42.5–58.2	122 (41.8)	36.2–47.6	201 (44.8)	40.2–49.5
Non-white	78 (49.7)	41.8–57.5	169 (58.2)	52.4–63.8	247 (55.2)	50.5–59.7
Head of household's education						
Under 9 years	81 (51.6)	43.7–59.4	144 (49.6)	43.8–55.5	225 (50.3)	45.6–55.0
10 years or more	76 (48.4)	41.0–56.3	157 (50.4)	44.5–56.2	223 (49.7)	45.0–54.4
Physical activity according to WHO recommendations (Global)						
Does not comply with recommendation	72 (45.9)	38.1–53.8	143 (49.3)	43.5–55.1	215 (48.1)	43.4–52.8
Complies with recommendation	85 (54.1)	46.2–61.9	148 (50.7)	44.9–56.5	233 (51.9)	47.2–56.6
BMI-for-age						
Without obesity	138 (87.9)	81.7–92.1	258 (88.5)	84.1–91.8	396 (88.3)	84.9–91.0
With obesity	19 (12.1)	7.8–18.3	33 (11.5)	8.2–15.6	52 (11.7)	9.0–15.1
Waist circumference						
Within recommendations	147 (93.7)	88.3–96.7	262 (89.9)	85.8–92.9	409 (91.2)	88.1–93.5
Above recommendations	10 (6.3)	3.3–11.7	29 (10.1)	7.1–14.2	39 (8.8)	6.5–11.9
Blood pressure						
Normal	144 (91.7)	86.2–95.1	228 (79.7)	74.5–84.0	372 (83.9)	80.2–87.1
High	13 (8.3)	4.8–8.4	58 (20.3)	16.0–25.4	71 (16.1)	12.9–19.7
Total cholesterol						
Within recommendations	142 (90.4)	84.7–94.2	246 (84.4)	79.9–88.1	388 (86.5)	83.0–89.4
Above recommendations	15 (9.6)	5.8–15.3	45 (15.6)	11.9–20.3	60 (13.5)	10.6–17.0
LDL-c						
Within recommendations	149 (94.9)	90.1–97.5	258 (88.5)	84.3–91.6	407 (90.8)	87.7–93.2
Above recommendations	8 (5.1)	2.5–9.9	33 (11.5)	8.2–15.7	41 (9.2)	6.8–12.3
HDL-c						
Within recommendations	76 (48.4)	41.0–56.3	137 (47.2)	41.5–53.0	213 (47.6)	43.0–52.3
Below recommendations	81 (51.6)	43.7–59.4	154 (52.8)	47.0–58.5	235 (52.4)	47.7–57.0
Triglycerides						
Within recommendations	110 (70.1)	62.3–76.8	185 (63.5)	57.8–68.9	295 (65.8)	61.3–70.1
Above recommendations	47 (29.9)	23.2–37.6	106 (36.5)	31.1–42.2	153 (34.2)	29.9–38.7
Obesity						
Absence	133 (84.7)	78.1–89.6	241 (82.8)	78.0–86.8	374 (83.5)	79.7–86.7
Presence	24 (15.3)	10.4–21.9	50 (17.2)	13.2–22.0	74 (16.5)	13.3–20.3
Dyslipidemia						
Absence	53 (33.6)	26.7–41.6	92 (31.6)	26.5–37.2	145 (32.4)	28.2–36.9
Presence	104 (66.2)	58.4–73.3	199 (68.4)	62.8–73.5	303 (67.6)	63.1–71.8
Cardiometabolic risk factors						
Absence	148 (94.3)	89.3–97.0	268 (92.2)	88.4–94.8	417 (93.0)	90.1–95.0
Presence	9 (5.7)	3.0–10.7	23 (7.8)	5.2–11.6	31 (7.0)	5.0–9.9
Continuous variables	ISA 2008		ISA 2015		ISA 2008 + ISA 2015	
	Mean±SD Median (IQR)		Mean±SD Median (IQR)		Mean±SD Median (IQR)	
Per capita family income per equivalent adult (R$)	789.2 (631.6)		847.0 (771.6)		816.1 (719.8)	
Diet quality index	59±6.0		63±6.8		61±6.7	
Weight (kg)	54.0 (14.0)		56.0 (17.5)		55.0 (17.0)	
Height (m)	1.63±0.1		1.62±0.1		1.63±0.1	

ISA, Health Survey of the municipality of São Paulo; 95 %CI, 95 % confidence interval; SD, standard deviation; IQR, interquartile range; BMI, body mass index; LDL-c, high density lipoprotein cholesterol; HDL-c, low density lipoprotein cholesterol. The data presented in this table refers to the absolute numbers of participants assessed and their respective proportions, not considering the sample design.

The most prevalent cardiometabolic risk factor was dyslipidemia (67.6 %; 95 % CI, 63.1–71.8), and low HDL-c alone had a prevalence of 52.4 % (95 % CI, 47.7–57.0). High blood pressure was observed in 16.1 % (95 % CI 12.9–19.7) of the adolescents, and obesity in 11.7 % (95 % CI 9.0–15.1). 7 % (95 % CI 5.0–9.9) had three or more risk factors concomitantly. Regarding lifestyle factors, the IQD-R mean was 61 points, and the proportion of adolescents who met the global physical activity recommendations was 51.9 % (95 % CI 47.2–56.6) (Table 1).

Demographic, quality-of-life, and biochemical variables were compared by sex, as presented in Table 2. The only characteristic that differed between males and females was height (*p* = 0.024), with females taller than males, though the difference was small (1.64 ± 0.1 m vs. 1.62 ± 0.1 m).

**Table 2:** Demographic, quality of life and biochemical characteristics according to gender in the ISA 2008 and 2015 adolescent population. São Paulo, Brazil^†^.

	Sex		
Variables	Male (*n* = 236)	Female (*n* = 212)	p-value
	n ( %)	n ( %)	
Individual's self-declared race/color			
White	107 (53.8)	92 (46.2)	0.564^a^
Non-white	125 (51.0)	120 (49.0)	
Schooling of head of household			
Up to 9 years	115 (52.3)	105 (47.7)	0.804^a^
10 years or more	116 (53.5)	101 (46.5)	
Physical activity according to WHO recommendations (Global)			
Does not meet recommendations	112 (52.6)	101 (47.4)	0.996^a^
Meets recommendations	121 (52.6)	109 (47.4)	
BMI-for-age			
Not obese	201 (52.3)	183 (47.7)	0.936^a^
Obese	27 (52.9)	24 (47.1)	
Waist circumference			
Within recommendations	204 (52.0)	188 (48.0)	0.317^a^
Above recommendations	23 (60.5)	15 (39.5)	
Blood pressure			
Normal	204 (54.3)	171 (45.7)	0.100^a^
High	32 (43.7)	41 (56.3)	
Total cholesterol			
Within recommendations	205 (53.3)	180 (46.8)	0.478^a^
Above recommendations	29 (48.3)	31 (51.7)	
LDL-c			
Within recommendations	210 (52.0)	194 (48.0)	0.423^a^
Above recommendations	24 (58.5)	17 (41.5)	
HDL-c			
Within recommendations	106 (50.0)	106 (50.0)	0.298^a^
Below recommendations	128 (54.9)	105 (45.1)	
Triglycerides			
Within recommendations	151 (51.5)	142 (48.5)	0.539^a^
Above recommendations	83 (54.6)	69 (45.4)	
Obesity			
Absence	192 (51.3)	182 (48.7)	0.201^a^
Presence	44 (59.5)	30 (40.5)	
Dyslipidemia			
Absence	68 (47.2)	76 (52.8)	0.117^a^
Presence	166 (55.2)	135 (44.9)	
Cardiometabolic risk factors			
Absence	210 (51.3)	199 (48.7)	0.078^a^
Presence	21 (67.7)	10 (32.3)	
Continuous variables	Mean±SD Median (IQR)	Mean±SD Median (IQR)	p-value
Family income per capita per adult equivalent (R$)	809.6 (623.5)	837.0 (822.6)	0.801^c^
Diet quality index	61±6.7	61±6.	0.839^b^
Weight (kg)	55.0 (16.0)	55.0 (18.0)	0.561^c^
Height (m)	1.62±0.1	1.64±0.1	0.024^b^*

SD, standard deviation; IQR, interquartile range; BMI, body mass index; LDL-c, high-density lipoprotein cholesterol; HDL-c, low-density lipoprotein cholesterol. ^†^ The analyses were conducted in survey mode, taking into account the study's sample design. ^a^ p-value obtained using the Chi-square test.

b p-value obtained using the *t*-test.

c p-value obtained using the Wilcoxon Rank-sum test.

Older adolescents were 19 % less likely (OR = 0.81; *p* = 0.008) to be obese and 26 % more likely (OR = 1.26; *p* = 0.004) to have a waist circumference above the recommended level. Compared with those in 2008, adolescents in 2015 had 2.07 times the likelihood (OR = 3.07; *p* = 0.006) of presenting high blood pressure, and 1.41 times the likelihood (OR = 2.41; *p* = 0.031) of presenting high LDL-c (Table 3).

**Table 3:** Associations of investigated cardiometabolic risk factors with age and sex in the adolescent population of the ISA 2008 and 2015. São Paulo, Brazil^†^.

Simple Logistic Regression Models									
	ISA-Nutrition (vs. ISA-2008)			Sex (vs. Male)			Age		
	OR	CI 95 %	p	OR	CI 95 %	P	OR	CI 95 %	p
High blood pressure	3.07	1.39–6.81	0.006*	1.86	0.95–3.65	0.068	1.38	0.81–2.34	0.231
Obesity	1.15	0.68–1.95	0.606	0.72	0.43–1.19	0.202	1.09	0.97–1.23	0.149
BMI-for-age obesity	0.94	0.52–1.73	0.854	0.98	0.54–1.75	0.936	0.81	0.70–0.95	0.008*
Increased waist circumference	1.67	0.77–3.64	0.194	0.71	0.36–1.40	0.319	1.26	1.08–1.48	0.004*
Dyslipidemia	1.10	0.73–1.67	0.642	0.73	0.49–1.08	0.118	0.94	0.86–1.04	0.216
High total cholesterol	1.75	0.94–3.26	0.076	1.22	0.71–2.10	0.479	0.99	0.87–1.12	0.845
High LDL-c	2.41	1.08–5.36	0.031*	0.77	0.40–1.47	0.424	1.00	0.86–1.16	0.985
Low HDL-c	1.05	0.71–1.55	0.811	0.82	0.56–1.19	0.298	0.99	0.91–1.08	0.811
High TG	1.34	0.88–2.04	0.166	0.88	0.60–1.31	0.539	0.99	0.90–1.08	0.784
CMRF without glycemia	1.38	0.62–3.09	0.425	0.50	0.23–1.09	0.083	1.16	0.98–1.38	0.086

*: statistical significance. Abbreviations: ISA, Health Survey of the municipality of São Paulo; OR, Odds Ratio; 95 %CI, 95 % confidence interval; p, p-value; LDL-c, high-density lipoprotein cholesterol; HDL-c, low-density lipoprotein cholesterol; TG, triglycerides; CMRF, Cariometabolic risk factors all the variables were categorized according to the cutoffs proposed by WHO, NHANES and Brazilian Guidelines on Arterial Hypertension. ^†^ Regression models were generated taking into account the study's sample design.

In 2015, adolescents were more likely (OR = 2.04; *p* = 0.011) to have high blood pressure compared to those analyzed in ISA 2008. Moreover, older adolescents were less likely (OR = 0.83; *p* = 0.024) to be obese, more likely (OR = 1.22; *p* = 0.027) to have a waist circumference above the recommended level, and were more likely to present with high blood pressure (OR = 1.17; *p* = 0.017). Individuals self-declared as non-white were less likely to meet the criteria for dyslipidemia (OR = 0.59; *p* = 0.018) and for elevated triglycerides (OR = 0.58; *p* = 0.012). Those whose heads of household had 10 years or more of schooling were less likely to have dyslipidemia (OR = 0.54, *p* = 0.004) and low HDL-c (OR = 0.65, *p* = 0.033). Adolescents who met the global recommendations for physical activity were less likely to have high LDL-C (OR = 0.48; *p* = 0.045) (Table 4).

**Table 4:** Associations of the investigated cardiometabolic risk factors with sociodemographic and lifestyle aspects in the adolescent population of the ISA 2008 and 2015. São Paulo, Brazil^†^.

Logistic Regression Multiple Models																		
	ISA-Nutrtition (vs. ISA-2008)			Age			Individual's self-declared color/race (vs. White)			Schooling of the head of household (vs. up to 9 years)			Physical activity (vs. Does not meet recommendation)			BHEI-R		
	OR	CI 95 %	p	OR	CI 95 %	p	OR	CI 95 %	p	OR	CI 95 %	p	OR	CI 95 %	p	OR	CI 95 %	p
High blood pressure	2.04	1.22–4.72	0.011*	1.17	1.03–1.33	0.017*	0.94	0.54–1.65	0.834	0.88	0.51–1.53	0.663	1.12	0.64–1.97	0.675	1.01	0.97–1.05	0.642
Obesity	1.11	0.63–1.96	0.725	1.08	0.95–1.22	0.247	1.10	0.64–1.87	0.730	0.78	0.46–1.31	0.347	0.92	0.54–1.57	0.772	0.99	0.95–1.03	0.535
BMI-for-age obesity	1.10	0.57–2.11	0.777	0.83	0.71–0.98	0.024*	1.48	0.79–2.77	0.224	0.86	0.47–1.58	0.629	0.89	0.48–1.65	0.721	0.99	0.95–1.04	0.729
Increased waist circumference	1.30	0.56–3.04	0.544	1.22	1.02–1.46	0.027*	0.52	0.25–1.09	0.081	0.58	0.28–1.21	0.148	0.83	0.40–1.74	0.622	1.01	0.95–1.07	0.779
Dyslipidemia	1.20	0.77–1.89	0.421	0.92	0.83–1.02	0.117	0.59	0.38–0.91	0.018*	0.54	0.35–0.82	0.004*	1.21	0.80–1.86	0.362	1.01	0.97–1.04	0.689
High total cholesterol	1.77	0.92–3.41	0.088	0.99	0.86–1.14	0.917	0.98	0.55–1.76	0.954	0.98	0.55–1.73	0.948	0.70	0.39–1.25	0.225	0.99	0.95–1.04	0.767
High LDL-c	1.99	0.86–4.60	0.107	1.02	0.86–1.20	0.840	1.27	0.63–2.56	0.506	0.87	0.44–1.73	0.696	0.48	0.23–0.98	0.045*	1.05	0.99–1.10	0.093
Low HDL-c	1.04	0.69–1.59	0.842	0.98	0.89–1.07	0.610	0.84	0.56–1.24	0.376	0.65	0.44–0.97	0.033*	1.27	0.85–1.88	0.239	1.01	0.98–1.04	0.584
High TG	1.51	0.96–2.38	0.074	0.97	0.88–1.07	0.559	0.58	0.38–0.89	0.012*	0.88	0.58–1.33	0.552	0.97	0.64–1.47	0.880	0.98	0.94–1.01	0.135
CMRF without glycemia	1.34	0.56–3.18	0.511	1.14	0.95–1.38	0.159	1.20	0.54–2.65	0.657	1.13	0.52–2.44	0.764	1.35	0.61–2.98	0.457	0.97	0.91–1.03	0.296

*: statistical significance. Abbreviations: ISA, Health Survey of the municipality of São Paulo; BHEI-R, revised diet quality index; OR, Odds Ratio; IC95 %, confidence interval of 95 %; p, p-value; LDL-c, high-density lipoprotein cholesterol; HDL-c, low-density lipoprotein cholesterol; TG, triglycerides; CMRF, Cariometabolic risk factors all the variables were categorized according to the cutoffs proposed by WHO, NHANES and Brazilian Guidelines on Arterial Hypertension. ^†^ All regression models were generated taking into account the study's sample design.

## Discussion

The findings of this study highlight a concerning cardiometabolic profile among adolescents living in São Paulo, marked by a high prevalence of dyslipidemia and a significant increase in elevated blood pressure between 2008 and 2015. Rather than reflecting isolated clinical phenomena, these findings likely mirror broader social, behavioral, and environmental changes experienced by adolescents in large urban centers over this period.

Dyslipidemia (67.6 %; 95 % CI 63.1–71.8 %) emerged as the most prevalent cardiometabolic risk factor, affecting more than two-thirds of the adolescents evaluated, with a prevalence similar to that reported in the nationwide cross-sectional study "*Estudo de Riscos Cardiovasculares em Adolescentes*" (ERICA), which found 64.7 % among adolescents aged 12 to 17 years19. Internationally, a US study using data from the 2011–2018 National Health and Nutrition Examination Survey (NHANES) found a dyslipidemia prevalence of 24.3 % among adolescents aged 12 to 19 [19]. Although similar prevalence levels have been reported in national studies such as ERICA, the magnitude observed remains striking when contrasted with data from high-income countries, particularly the United States. These differences may be partially explained by dietary patterns characterized by high intake of sugar-sweetened beverages and saturated fats, which have expanded substantially in Brazil over the last decade. In parallel, reductions in habitual physical activity and increased sedentary behaviors may further contribute to unfavorable lipid profiles during adolescence, a critical period for metabolic programming.

The prevalence of low HDL-c in the population of this study (52.4 %; 95 % CI 47.7–57.0) was higher than the national prevalence (46.8 %; 95 % CI 44.8–48.9) and that of the Southeast (45.9 %) [20]. In the Population-Based Health Survey of the Municipality of Campinas - SP (ISA Camp 2014/15), the prevalence of adolescents with low serum HDL-c concentrations was 41 % [21]. Meanwhile, studies conducted using NHANES data and its various cycles have shown lower prevalence rates than those in Brazil, ranging from 8 % to 25 % [[22], [23], [24]]. The comparative analysis of Brazilian adolescents and those of Latin American origin living in the USA corroborates these findings, given the prevalence of 41 % (95 % CI 33.1–48.9) of low HDL-c among Brazilian adolescents and only 13.8 % (95 % CI 10.9–16.7) among those of Latin American origin living in the USA [25]. This finding underscores the central role of environmental and behavioral determinants, rather than ethnicity per se, in shaping lipid profiles.

The prevalence of high blood pressure in this study (16.1 %) showed greater fluctuations compared to national and international data. ERICA reported a prevalence of 24 % in Brazil and the Southeast, which is almost 50 % higher than that reported in this study. In the US NHANES population, the prevalence was approximately 12 %, lower than both this study and the Brazilian population. New guidelines and updated research have influenced these risk factors. The increase in high blood pressure observed in 2015 may reflect broader lifestyle changes over the period, including reduced physical activity levels, increased consumption of unhealthy foods, and greater exposure to urban stressors, rather than isolated biological changes.

Data from the HBS (Household Budget Survey) 2008–2009 and ERICA 2013–2014 indicated a growing national prevalence of obesity in adolescents: 4.9 % and 8.4 %, respectively [20,26,27]. In the Southeast, the prevalence of obesity in 2016 was 8.6 % [20]. A study evaluating the trend in the prevalence of obesity in the city of São Paulo, based on data from the 2003, 2008 and 2015 editions of the ISA, identified prevalence rates of 3.7 % (95 % CI 2.3–5.9) in 2003, 5.9 % (95 % CI 4.1–8.5) in 2008 and 9.3 % (95 % CI 7.6–11.4) in 2015, data that expresses that the prevalence has been maintained, according to the projection of the confidence intervals [25]. Global data from the WHO also points to an increase in the prevalence of obesity in adolescents [11].

Regarding the presence of three or more concomitant CVRMs, the data from this study showed a higher prevalence (7.0 %; 95 % CI 5.0–9.9) among São Paulo adolescents, compared to the ERICA study for the Brazilian population (2.6 %; 95 % CI 2.3–2.9) and the Southeast region (2.4 %; 95 % CI 1.9–3.0). The ERICA study used higher cut-off points for blood pressure (≥ 130/≥ 85 mmHg) and plasma triglycerides (≥ 150 mg/dL), and did not consider BMI or total plasma cholesterol in the classification of CVRF [28]. Globally, The Lancet reported an estimated prevalence of three or more concomitant CMRFs of 4.8 % among adolescents evaluated (95 % CI 2.9–8.5) in a systematic review and meta-analysis study [1].

Another factor that may explain differences in the prevalence of CVRM among adolescents across studies is the classification criteria used. In the present study, the authors adopted the CVRM proposed by NCEP-ATP III [18]. for adolescents, primarily because there is no consensus on which criterion best fits the adolescent population. Additionally, it is straightforward to apply in epidemiological studies, as it does not require measuring insulin resistance or abdominal obesity. The NCEP-ATP III criterion is based on the presence or absence of three or more risk factors, with updates to recommendations for each risk factor provided by Brazilian public health authorities. The cut-off points for the study population were divided by age group, when applicable [18].

In 2015, adolescents in this study were more likely to have high blood pressure than those in the ISA 2008 (OR = 2.04; *p* = 0.011). The SBC confirms the increase in rates of high blood pressure and hypertension in adolescents in recent years14. Older adolescents had a higher likelihood of having high blood pressure (OR = 1.17; *p* = 0.017) in this study, corroborating the 2020 Brazilian Hypertension Guidelines15. These findings include results from a systematic review for the US Preventive Services Task Force, indicating an increase in prevalence with age in adolescents (*p* < 0.001) [29].

A difference in height-for-age was observed between sexes, with females being taller than males at age 12; however, this trend reverses by age 19, when males become taller. The intersection point occurs around 13 years and 1 month, according to WHO growth curves. These age-related differences observed in this study further support the influence of developmental and behavioral transitions. Older adolescents were less likely to present obesity but more likely to have increased waist circumference, reflecting physiological growth and pubertal changes combined with shifts in body fat distribution. This pattern highlights the limitations of BMI as a sole indicator of cardiometabolic risk during adolescence and underscores the relevance of central adiposity as a more sensitive marker.

The results of this study showed that older adolescents were less likely to present obesity (OR = 0.83; *p* = 0.024), yet more likely to have a waist circumference above the recommended level (OR = 1.22; *p* = 0.0027). These findings are consistent with data from the 2008–2009 HBS, which reported a higher prevalence of obesity in younger adolescents, at 8.6 % among those aged 10– 11 years, compared to 3.7 % in the 18– 19-year-old group [27]. Similar trends were observed in ERICA study, where among adolescents aged 12 to 14 years, obesity prevalence reached 8.5 % (95 % CI: 7.7–9.4) in females and 10.8 % (95 % CI: 9.7–12.0) in males, whereas among those aged 15 to 17, prevalence dropped to 6.7 % (95 % CI: 5.8–7.7) and 7.3 % (95 % CI: 6.6–8.1), respectively. In the Southeast region, a similar pattern was observed [20].

Although obesity tends to decline with age, waist circumference increases during adolescence due to normal growth and pubertal development [3,13,17]. The ISA-Capital 2008 study showed that adolescents with excess weight had higher frequencies of altered lipid profiles, particularly low HDL-c and high triglyceride levels, despite a low overall prevalence of dyslipidemia. This highlights the need for preventive actions targeting modifiable risk factors during this critical stage of life [30].

Individuals who declared themselves as non-white were less likely to have dyslipidemia (OR = 0.59; *p* = 0.018) and to have TG above recommendations (OR = 0.58; *p* = 0.012), suggesting a protective factor for this population. This might be due to the relationship between color/race as a socioeconomic and political construct that overlaps with biological or genetic features, as presented here in serum lipid levels. In agreement to that, a population-based study showed higher odds of having dyslipidemia (OR = 3.71; *p* < 0.01), low HDL-c (OR = 2.81; *p* < 0.01), high LDL-c (OR = 1.24; 95 %CI 0.77–1.99) and high TG (OR = 1.67; 95 %CI 0.64–4.32) in high school adolescents (14 to 19 years old) from public schools compared to private schools [31].

The analysis revealed that shifts in the prevalence of cardiometabolic risk factors from 2008 to 2015 were solely evident for elevated blood pressure, with no sex-based variations. Nevertheless, while most cardiovascular risk factors (CVRFs) did not exhibit statistically significant differences between the two examined periods, their overall prevalence remains clinically essential, particularly dyslipidemia, as previously noted. This necessitates ongoing surveillance and targeted interventions, given the well-documented association between adolescent cardiovascular and metabolic risk factors and their substantial impact on cardiovascular disease (CVD) risk, morbidity, and mortality in later adulthood [32].

A beneficial strategic measure for the prevention, treatment, and maintenance of health, including the reduction of dyslipidemia and CVD, such as hypercholesterolemia, is the practice of physical activity, strongly recommended by various public health authorities, such as the WHO, SBC, and Brazilian Society of Pediatrics (SBP)3,15,16. The claimed benefits can be observed in the present studies: adolescents who met the WHO global physical activity recommendations were 52 % less likely to have elevated LDL-c (OR = 0.48; *p* = 0.045).

This study has limitations. First, information on Tanner stages of sexual maturation was not available, which may affect the interpretation of anthropometric and lipid alterations during adolescence. In addition, fasting glucose was not measured in ISA-Nutrition 2008, preventing the inclusion of the glycaemic component in the NCEP-ATP III criteria for classifying FRCM. Comparisons between 2008 and 2015 should also be interpreted cautiously, as the surveys represent independent cross-sectional samples with different sample sizes. Although dietary intake was assessed using 24-hour recalls and diet quality scores, more detailed analyses of dietary patterns were beyond the scope of this study. Socioeconomic status was captured through proxy indicators such as household education and income, and the possibility of residual confounding cannot be excluded. Finally, physical activity was self-reported, which may have introduced measurement error. Despite these limitations, the study provides population-based and clinically measured cardiometabolic data from a representative sample of adolescents in São Paulo, enhancing comparability with other studies and relevance for public health monitoring.

The study highlights the high prevalence of cardiometabolic risk factors among adolescents in São Paulo, particularly dyslipidemia. Significant changes in the prevalence of high blood pressure were observed over the two years studied. The findings underscore the importance of implementing preventive and interventional strategies tailored to these adolescent groups to reduce the risk of developing current and future cardiometabolic diseases.

## Study funding sources

This work was supported by the São Paulo Municipal Health Department (grant number #2013-0235936-0), Research Support Foundation of the State of São Paulo (grant numbers #2012/22113-9, #2017/05125-7), National Council for Scientific and Technological Development (grant number #303904/2021-6) and Coordination for the Improvement of Higher Education Personnel for Scholarship (Financial code 001).

## Data availability

The data that support the findings of this study are available from the corresponding author.

## Conflicts of interest

The authors declare no conflicts of interest.
